# South African managers in public service: On being authentic

**DOI:** 10.3402/qhw.v9.20630

**Published:** 2014-01-15

**Authors:** Antoni Barnard, Nirvana Simbhoo

**Affiliations:** Department of Industrial and Organisational Psychology, University of South Africa, Pretoria, South Africa

**Keywords:** Authenticity, authentic leadership, identity work, psychological well-being, interpretative phenomenology, hermeneutic phenomenology

## Abstract

South African managers in public service consistently face challenges related to managing a well-adjusted and productive diverse workforce. Following the notion that leadership authenticity fosters positive psychological employee capacity, the aim of this study was to explore the meaning essence of authenticity as lived in the work–life experiences of senior managers in public service. Five senior managers in public service were purposefully selected based on their articulated challenges with being authentic at work, whilst attending a diversity sensitivity workshop. From a hermeneutic phenomenological perspective, in-depth interviews were used, and an interpretative phenomenological analysis yielded two predominant themes offering a description of what it means to be authentic. Authenticity is experienced as an affective state that results from a continuous self-appraisal of the extent to which expression of self is congruent with a subjective and socially constructed expectation of self in relation to others. Authenticity seems to develop through a continuous process of internal and external adaptation, and it leads to ultimately building a differentiated yet integrated identity of self. A reciprocal dynamic between feeling authentic and self-confidence alludes to the potential importance of authenticity dynamics in identity work.

As the largest single employer in South Africa, the Public Service faces numerous challenges, as do its employees. Complexities associated with increasing workforce diversity, service inefficiency, and distrust in leadership have been identified as critical challenges in maintaining a healthy and productive workforce (Public Service Commission, 2007, 2008). In developing leadership capacity, senior managers in a provincial government sector participated in a diversity management project which included attending diversity sensitivity workshops. During these workshops, it became apparent that several managers struggled with authentic self-expression when managing diverse employees in a context driven by a sometimes contradictory political supremacy versus a focus on service to the people.

Authentic leadership has been defined as a pattern of leader behaviour that promotes and fosters positive psychological employee capacity and ethical work behaviour (Walumbwa, Avolio, Gardner, Wernsing, & Peterson, 2008). Leaders’ authenticity is thus linked to employees’ psychological well-being, and it is suggested to promote constructive interpersonal work relationships and improve team functioning and productivity (Hannah, Walumbwa, & Fry, 2011). Research interest in authenticity at work has demonstrated the destructive effect of inauthentic feelings and behaviour on work satisfaction and employees’ sense of well-being (Vannini, 2006). Lowered self-esteem and interpersonal conflict are linked to being inauthentic (Carson & Langer, 2006; Goldman & Kernis, 2002; Kernis, 2003) and to the potential to lead to psychopathology through self-alienation (Wood, Linley, Maltby, Baliousis, & Joseph, 2008). Neff and Harter (2002) conclude that authenticity and true self-expression are necessary for optimal psychological health. Authenticity is also positively associated with motivation and well-being at work (Kifer, Heller, Perunovic, & Galinsky, 2013; Ménard & Brunet, 2011; Ryan, LaGuardia, & Rawthorne, 2005). In fact, according to Wood et al. (2008), authenticity is regarded as the most fundamental aspect of well-being and healthy functioning in many mainstream psychology perspectives.

Various researchers have indicated a paucity of empirical research in authenticity (Lopez & Rice, 2006; Wood et al., 2008), and they criticize the resulting lack of conceptual clarity and a coherent body of knowledge in this regard (Harter, 2005). Vannini (2006, p. 236) states that “despite their significance for the self and society, the concepts of inauthenticity and authenticity have long suffered from definitional lack of clarity,” and that “no qualitative research exists on what it means to feel authentic or inauthentic.” Authenticity work has been built from humanistic, existentialist, and psychodynamic psychology perspectives, including the work of Freud, Jung, Frankl, Erikson, Maslow, Rogers, Buhler, Perls, and James Bugental (Rahilly, 1993). Positive psychology more recently claimed the authenticity construct as a basic human strength (Peterson & Seligman, 2004) and encouraged a resurgence of interest in authenticity (Wood et al., 2008). Lopez and Rice (2006) call for continued study in authenticity, as such a focus may illuminate interpersonal conditions and processes that promote or inhibit self-expression. Moreover, authenticity inquiry should, according to Erickson (1995), focus on the subjective experiential process of authenticity construction and on those aspects that underlie feelings of inauthenticity for the individual.

Exploring the authenticity experiences of managers in public service may be valuable in developing a leadership context conducive to employee well-being in general. Understanding how these managers experience being authentic may contribute to creating an authenticity-enabling working environment and relevant training and self-development initiatives for managers.

## Research objective

The aim of this study is to develop a deeper understanding of the meaning of authenticity as experienced by senior managers in provincial government. In this regard, the following research question was formulated: How is being authentic experienced by senior public service managers?

## Method

Authenticity was studied here from a hermeneutic phenomenological orientation and through an interpretative phenomenological analysis of transcribed narrative interviews. Hermeneutic phenomenology is an ideographic method of qualitative inquiry focussed on interpreting individuals’ experiential accounts to obtain a critical understanding of the phenomenon under study (Kawulich & Holland, 2012; Shaw, 2010). A hermeneutic phenomenological orientation incorporates scientific assumptions underlying both phenomenology and hermeneutics and attempts to uncover the deeper meaning of a phenomenon, a meaning that lies below the surface subjective experiential accounts thereof (Kafle, 2011).

Aligning our scientific orientation to critical realism as well as the assumptions underlying social constructionism, we believe in a perspectival reality that is socially constructed, that can only be accessed through subjective experience, and that is undeniably influenced by our own perspectives. Ontologically, we therefore support the importance of studying authenticity from a subjective first-person account, as personal well-being and authenticity states are ultimately self-experienced (cf. Erickson, 1995; Fleeson & Wilt, 2010). Congruent to Heidegger's hermeneutic or interpretative phenomenology, rather than suspending our preconceptions we believe that these play an inevitable part in our interpretation of the data (see Gearing, 2004; Norlyk & Harder, 2010). As such, we have endeavoured to explicate our preconceptions by employing reflexive strategies (personal and theoretical) (see Gearing, 2004) to facilitate a critical attitude towards the research phenomenon. We have endeavoured to describe the meaning of authenticity, based on an exploration of intersubjective truth and shared human experience, seeking coherence between and integration of the participants’ lived experience and an independent reality (theory and our preconceptions) in the research findings (cf. Norlyk & Harder, 2010; Snape & Spencer, 2004).

### Who are we? Explicating researcher roles and preconceptions

In line with the reflexive practice of making who we are explicit, introducing ourselves as researchers is essential. The second author, an Indian female working as a human resource management and development manager in provincial government, conducted this research for her Industrial Psychology Master's degree. Her involvement in a diversity management project for senior public service managers augmented her empathic experience with several of the management staff, and their ability “to be themselves” as leaders of diverse work teams sparked her interest to study authenticity. Her insider role provided a unique advantage to understanding the context in which the research was conducted, as well as a constant conscious challenge to remain aware of subjectivities potentially influencing research results.

The first author is a White female academic and industrial psychologist who supervised the master's study and in her own research focusses on employee well-being and its relation with various existential constructs such as authenticity. The *a priori* knowledge of the first author subsequently impacted various aspects throughout the study and became more predominant post data gathering and during data analysis. Both authors were involved as facilitators of the diversity sensitivity workshops run as part of the overall diversity management project. Our preconception was that managers’ ability for authentic self-expression facilitates effective leadership in a diverse team setting as it creates a personal sense of wellness and open, trusting interpersonal relationships with subordinates and peers.

### The research context

The Public Service is a large, complex bureaucratic organization with a culturally diverse workforce. It is characterized by both structural and people-related challenges. Structurally, political and administrative leadership coincide to achieve the government's political mandate as well as its core service to the public, creating a unique management and reporting hierarchy. Fraud and corruption, a lack of trust, poor leadership, management ineffectiveness, poor communication, and performance anxiety are some of the reported challenges noted in the Public Service (Public Service Commission, 2007, 2008). These problems, which are experienced throughout the Public Service, raise the question of leadership ethics, integrity, honesty, and authenticity.

From a people perspective, employees work within a political environment that is significantly different from that of a profit-driven organization. In a political environment, they are exposed to power relationships, political agendas, and factions challenging the trust and transparency in relationships. Power relationships are established and used to further individual aims, often at the expense of others and service delivery. Leadership effectiveness and free self-expression are often compromised in this way. Employees are often forced to turn to the grievance procedure to resolve conflicts, implying that other dispute resolution mechanisms have broken down and also suggesting a breakdown in relationships. The cultural diversity in the province is mirrored in the workplace and exacerbates interpersonal tension because strong political factions are historically deeply manifested along racial boundaries. We have witnessed high levels of conflict on an interpersonal level, resulting in high levels of stress and poor performance.

The Public Service is furthermore critically challenged in the area of fraud and corruption. The numerous cases of fraud and corruption reported daily in the media support a public perception of a fraudulent and corrupt Public Service. Nepotism, deceit of others, secrecy, imposter tendencies, and self-deception are examples of inauthentic self-behaviour (Harter, 2005), and exposure to such behaviour without recourse or opportunity to report contributes to interpersonal conflict, creates negative emotions, and impacts one's ability to act authentically.

### Participants

Two males and three females, with ages ranging from 35 to 45 years, participated in this study. Participants were identified through the purposive sampling (Laher & Botha, 2012) of senior-level managers in charge of a diverse work group in a provincial Public Service department. All of these managers at the time participated in a 2-year diversity management project as part of their leadership development. The researchers approached senior managers based on variation in their gender, race, and seniority, as well as on the extent to which “being themselves” was apparent in the narratives that they presented during diversity sensitivity workshops run over the course of 2 years; transcripts from the diversity sensitivity workshops were scrutinized to identify narratives reflecting a personal concern with authentic self-expression. Five managers agreed to participate in the research. The decision to participate was potentially influenced by work load as well as the politically sensitive nature of the work environment. Finally, the sample included senior-level managers of teams with between 5 and 10 members, and their managerial experience varied from 8 to 15 years. Three managers were Indian, one was Mixed Race, and one was African. At the time, no White managers participated in the diversity sensitivity workshops.

### Data collection and recording

Data were gathered through in-depth narrative interviews, in which interviewers posed open-ended questions and allowed continued exploration of participant responses to uncover the experience of authenticity in the sample context. Commencing with the first two interviews, we gathered that authenticity either is not a conscious experience or is an uncommon construct, resulting in managers feeling very uncertain as to what they should relate. To address this uncertainty, we moved to first ask managers to describe what authenticity means to them. The question seemed to ease them into a more conscious awareness of authenticity, and we could then focus on asking them the primary question, which was to narrate work situations in which they experienced being authentic or inauthentic. Follow-up questions probed their narrations to reveal more in-depth contextual aspects of being or feeling authentic. Interviews were digitally recorded, as agreed with each manager prior to commencing the interview.

### Analysis of the data

Supporting van Manen (1990), Kafle (2011) emphasizes that there is no fixed set of prescribed methods to conduct hermeneutic phenomenological research. We were guided by the step-wise thematic structural analyses proposed by Lindseth and Norberg (2004) and Kawulich and Holland (2012) within the framework of interpretive phenomenological analysis (IPA). Congruent to the philosophical notions of hermeneutic phenomenology, IPA assumes a critical realist ontology as it aims to understand what a phenomenon means through studying experiential accounts thereof (Shaw, 2010). Interviews were transcribed verbatim, and the first step in the analysis process entailed familiarization and immersion in the data through naïve reading and rereading of transcriptions to gain a holistic understanding of the text.

Next, we followed a thematic structural analysis of the text with the aim of formulating themes that would confirm our initial understanding of the managers’ experiences. As a point of departure in the thematic structural analysis, significant statements directly related to each manager's lived experience of authenticity were marked by using the research objective and question as a guide (cf. Marshall & Rossman, 2006) and by constantly comparing it to our initial naïve reading (cf. Dale, Söderhamn, & Söderhamn, 2012; Lindseth & Norberg, 2004). The identified statements were then rephrased to reflect the underlying meaning of each statement, and thus formulations of meaning (see Kawulich & Holland, 2012) were constructed and expressed in everyday language (cf. Lindseth & Norberg, 2004). Subthemes were then developed by clustering similar meaning formulations. These subthemes were further clustered and then abstracted and condensed into themes describing what it means to be authentic in this research context. In the final analysis phase, we attempted to describe what it means to feel authentic, remaining very much aware that our interpretations were related to the context and perspective of senior public service managers in a provincial government work sector.

### Ethical considerations

Informed consent was proactively obtained from every manager as well as from the employing organization. Anonymity and confidentiality of participants and of the employment organization were ensured throughout the study. Managers were further informed of their right to withdraw from the study at any time. The Human Resources Manager gave written permission to conduct the study on behalf of the employing organization. Consent was given to keep the identities of the five research participants anonymous. The study was also approved by the University of South Africa (UNISA) Department of Industrial and Organisational Psychology, and the research was conducted in alignment with the Ethical Rules of Conduct for Practitioners registered under the Health Professions Act, 1974 (Health Professions Council of South Africa, 2006) as well as with the Policy on Research Ethics of UNISA (UNISA, 2007).

## Findings

### Naïve reading

Through the naïve reading of the text, we were left with the impression that senior managers in public service experience authenticity when they feel good about themselves in work-related interactions as a result of being able to be and act in accordance with their personal values and in accordance with their preferred personality styles. The experience of being inauthentic was related to negative emotions resulting from situations where they had to act or speak differently from what they felt comfortable with or from how they think they should have acted or spoken. These negative emotive consequences were linked to not being able to be themselves and to feeling inauthentic. Personal inauthenticity was then rationalized externally and internally by ascribing it to conflict between external and internal expectations of self. It seems as if managers are frequently busy with impression management in the work context and an adjustment of the self with regard to various work–life role expectations.

### Thematic structural analysis and understanding

For us, the managers’ description of what it means to experience authenticity is captured in two essential themes, each with sub-themes elaborating on our understanding of what it means to be or feel authentic in this research context. The first theme describes the authenticity experience as a subconscious, emotively driven appraisal of self-congruence in relation to others. With the second theme, we understand authenticity as a socio-psychological process through which managers address the existential question “Who am I?” by differentiating the self as unique yet also integrating normative expectations of the self in context. Table I provides examples of the data analysis showing how verbatim extracts were meaningfully formulated and clustered into subthemes and themes.

**Table I:** Example of data analysis content and process.

Verbatim	Formulation of meaning	Sub-theme	Sub-theme clusters	Theme
I have confidence that I am doing the right thing.	Acting in accordance with my belief system	Being congruent to one's belief system	Congruent expression of self	An intuitive, emotively driven appraisal of self-congruence in relation to others
People have an opportunity to understand and experience me as I am and as nothing else.	Expressing myself as I am	Expression of self		
It's things that you are asked to do that you feel that these are actually not right, I would not approve of this.	Am I doing the right thing?	Subjective appraisal process	Subjective appraisal of self-congruence	
I am quite comfortable with who I am because I think that it is appropriate.	Feeling I am appropriate	Subjective appraisal process		
In some cases I have been quite upset, but you can't hold it in you and you find ways to deal with it, like saying that well, there is nothing I can do, so just let it go.	Emotional discomfort brings feeling inauthentic to consciousness	Emotional process	A subconscious, emotively driven dynamic	
You've got to understand what your role is so for somebody who has not actually understood that then it becomes difficult to adapt to this and hence for you while you want to remain who you are but first you need to adapt to the changed circumstance and then be in a position to do what is expected.	Understanding one's different roles and adapting to different contexts	Adjusting to role and context expectations	A continuous process of internal (role) and external (context) adjustment	A differentiated yet integrated self in context
The work environment, like I've said, has been characterized to a certain degree by politics which has meant that there've been dubious appointments which also affects us, for example, and also impacts on the mobility potential I think for people down there, those are the things	Political agendas complicate authentic expression	Political context inhibits trust inhibits authenticity		
I think, I am myself all the time, but the kind of person that I am at work in like this professional work environment is different from when I am with my family and friends.	Having different roles to play in your life yet remaining authentic	Integrate different roles without losing core self	Integrating multiple roles	
I found that, that practice persisted in this department because certain people had perceptions of you and they labelled you and this is what you were and this is how you were and because you were uncertain of yourself and how you should be you just accepted that	Being uncertain of oneself hinders authenticity experience	Self-confidence and authenticity	Self-confidence facilitates authentic self-expression, and that again confirms and builds self-confidence	
I could've left my job you know that kind of feeling but obviously my need for security prompted that I stay. I mean not just walk away, …, I think maybe the impact on me was that a lack of self-confidence and of self-esteem, maybe I wasn't leading in managing as properly as I could because of that and because of the uncertainty he placed on me	Lack of self-confidence and self-esteem hinders authenticity experience	Self-confidence and authenticity		

#### A subconscious, emotively driven appraisal of self-congruence in relation to others

Our interpretation of managers’ narratives led us to describe the experience of authenticity as a continuous, intuitive self-appraisal process of the extent to which one is able to express the self in congruence with how you expect to be in relation to others. Being authentic truly seems to be a process rather than an end state, and it incorporates a consistent cognitive and affective self-appraisal of self in interaction with others. The self-appraisal process is a natural, intuitive dynamic that does not necessarily happen consciously, and it has an emotive consequence manifesting as either dissatisfaction with self or a positive sense of self. The stability of the emotive consequence fluctuates as the person continues to re-evaluate the self in different work–life contexts throughout the life span. Authenticity manifests as a transient affective state that results from one's positive appraisal of self-congruence in relation to others. As such, we found authenticity to be an affirmation of the self and inextricably a part of identity work. Subthemes include a congruent expression of self, a subjective appraisal of self-congruence, and a subconscious and emotively driven dynamic.

#### Congruent expression of self

For the managers, their authenticity experiences were predominantly related to situations where they could express themselves in a congruent manner. Self-expression and self-congruence seemed to be keys to how authenticity is experienced, and *congruent* self-expression was implied in various ways such as being congruent to one's sense of true self, being congruent to one's belief systems, and being consistent in one's behavioural style.

For managers, being authentic fundamentally means to be able to express oneself in a way that reflects a unique and true self, and words such as being *original*, *real*, and *true to self* were repeatedly used in managers’ narratives. In describing feeling authentic, two managers likened authenticity to “originality” and referred to their behaviour as reflecting individual uniqueness as opposed to feeling very dissatisfied when they felt they had to follow conventional behaviour and decisions displayed by others in the department. One equates feeling authentic to “just being real, who you really are” when he was able to express his concerns about a work situation he did not feel comfortable with. His/her superior allows him/her to openly express his opinion and apply his/her own style in managing disagreements amongst staff. He/she is thus able to live what he/she believes is right, as opposed to a previous scenario in his/her life where he/she had to denounce his/her way of doing things, leaving him with a feeling of consistent internal dissonance, in which he/she remembered his/her yearning for “something that is the actual thing: it is a real representation of that type of feeling, a true representation of my personality and what I actually feel.” Being congruent to what one believes is right or wrong, and appropriate or inappropriate, is therefore also described as essential to feeling authentic.

Some managers related situations in which they emphasized being able to behave consistently across time and context, for example: “My behaviour today and responses today to a similar problem will be quite similar if the same problem is raised on another day.” For us, congruence is implied here in being able to express oneself in one's behaviour over time in a manner that is congruent to the person you feel you are. Consistency of self over time and across situations was described by some managers as characteristic of their authentic expression, especially in a relational context.

The effect of a lack of authenticity is described as

For me it would generally lower their morale because sometimes you are preaching something to them at one stage and then you find that you again are saying something different to what you have said; for them the issue is that they've got to stick to the rules,

as opposed to

What consistent behaviour does, my personal assessment is that it creates comfort and by creating comfort, people themselves feel free to be authentic with you and what people also understand is that they understand the parameters within which you would act, in the way you would express yourself.

#### Subjective appraisal of self-congruence

We thought that the notion of self-congruence presupposes a subjectively predefined true or real self, and we explored the narratives for clues as to what the true or real self entails. Apart from managers using related words (as noted in the previous subtheme), our clues derived from managers’ narratives about being authentic when they feel that they are acting “right” or appropriately, “I am quite comfortable with who I am because I think that it is appropriate.” The root of the appraisal therefore seemed subjective to us and defined by an underlying normative system, as was also alluded to by another manager: “I think by acting authentically at my level, I have confidence that I am doing the right thing.” Some managers spoke of authentic behaviour as being valid or correct, referring to being true to their subjective sense of what is appropriate in a certain context:

It would be something that, well, is correct, but also valid, valid in terms of the context you were using it in, for authentic it must be absolutely correct, but I think that I would extend it to look at the circumstances that you were in for it to be truly authentic.

As such, evaluating themselves as right or appropriate in a non-definitive manner led us to deduce that what is regarded as right and appropriate is ultimately subjectively defined, yet also socially constructed.

Managers’ self-critique in acting appropriately or correctly when they felt authentic alludes to an appraisal of the self in terms of a normative or ideal self. Being authentic is experienced as being true to the “real” or “true” self, yet the construction of the self seems to reflect an individual's sense of how he or she *ought to be* as reflected in a personal value system that derives from being brought up in a specific social context. Managers invariably referred to their struggle to be authentic in the work context due to being labelled, judged and stereotyped. For us, their concern with external impressions with regard to their authenticity furthers the notion that the true or real self is embedded in their social identity construction. Moreover, one of the managers exclaimed,

My authenticity has its basis in my life experiences, it has its basis in my upbringing, it has its basis in my morals and my morality, and I think largely my authenticity has its basis in my religion and spirituality.

#### The authenticity appraisal is a subconscious, emotively driven dynamic

The appraisal dynamic discussed above shows a strong connection with the individual's emotions. In her strive for authenticity, a female manager says, “I try to achieve it but it's not always easy, it is something that can torture me sometimes.” Similarly, for another the inability to authentically self-express creates anger and frustration: “It also creates so much pressure, there are ways to do things and achieve them easily … that frustrates me, you can't say anything about it.” Their experiences of inauthenticity manifest in emotional discomfort which creates a conscious psychological tension to stabilize feeling authentic again: “you have got to find a way to deal with this, you can't let things eat you up that you can't even focus on what you are doing,” and

in some cases I have been quite upset, but you can't hold it in you and you find ways to deal with it, like saying that well, there is nothing I can do, so just let it go.

Managers all unawares strive to feel authentic because they want to avoid feelings of self-dissatisfaction in being able to express themselves in a self-congruent manner when in relation with others.

#### A differentiated yet integrated self in context

This is a continuous process of internal (role) and external (context) adaptation. All of the managers spoke about feeling authentic in contextually relative terms. Being authentic for them entails adjusting to different role and context expectations and integrating these into an acceptable self. One explained that “sometimes one may not be fully authentic, but try to be as authentic as possible.” For us, this meant that one's authenticity is contextually dependent as one cannot be the same in different contexts, with different people, or in different roles, as was also evident when one manager reflected on his ability to be different at home and at work:

You can't manage your family and friends so you have to be different and treat them differently, plus, you know, your family, you love them, you don't necessarily love the people you work with in the same way and I think your love for your family causes you to be different and accept them.

Managers described experiencing authenticity despite adjusting to external expectations. The extent of their adjustment seems to have been determined by them valuing their adjusted behaviour to be necessary, and thus they remain congruent to what they deem important and appropriate, that is, their internal value system. Another female manager narrates how she sometimes feels that her subordinates do not accept her as an authority because they hold certain perceptions of how women should conduct themselves. Although she attempts to adjust to these expectations, she sometimes finds herself “in trouble” when she's not conforming to their notion of being feminine in her work context:

In the current context it's difficult to be the real you because there are other factors that one has to consider but by and large I always feel that I'm the real me, sometimes it is that real me that puts me into trouble because it's difficult to be somebody else but what I try and do is to try and understand the situation and adapt to it.

Her being consciously aware of adjusting to external expectations and believing in the necessity thereof make her comfortable with the fact that she remains authentic and true to herself.

Adjusting to role and context expectations in a politically structured and diverse work environment holds particular challenges for authentic self-expression, as is evident in the struggle for acceptance of her authority experienced by the aforementioned female manager. Managers are consistently confronted with power relationships and political agendas impacting their inter-relational trust and, consequently, their willingness to be transparent in their self-expression. Another manager relates how the political work environment creates barriers to his authentic self-expression. He experiences a lot of uncertainty and does not know who to trust, causing him to consistently manage impressions to the extent that he does not feel he can freely be his true self:

I think for a long time it was very stressful being in this political environment and all of the pressures that go with it … you still face that instability and insecurity in the environment … and that the office changes all of the time and you don't know how it can impact on you.

Conflict and distrust make one guarded, and being able to express oneself openly and freely only seems to come more easily when you too are in a beneficial power position. Freedom seems to come with promotion and seniority and allows one of the female managers greater comfort in being herself:

working under circumstances there where people give you more space and allow you to do the things that you need to do you actually start to do things that you want to do and be yourself.

In this process of internal (role) and external (context) adjustment, authenticity seems to consistently evolve as one matures and finds balance between external role and context expectations and the true self. As such, authenticity may not be a stable disposition. It fluctuates in tandem with one's adjustment, yet it may become more stable as one matures and/or when authority and power positions sway in one's favour.

#### Integration of multiple selves

Linking to role and contextual adjustment as key to one's authenticity dynamics is the ability to not only adjust to multiple role expectations but also authentically integrate multiple roles as part of the self. When we asked managers in the study to describe a time when they could truly be themselves, they reported a need to play differing roles in various circumstances. Authentic self-expression in different roles came across as fairly commonplace and a natural part of how the self evolves and matures:

I think, I am myself all the time, but the kind of person that I am at work in like this professional work environment is different from when I am with my family and friends,

and

I think now, I am more myself, but sometimes, especially if there is conflict and people problems then you have to, well you don't have to be different or like someone else, but you have to play a different role, be more firm, stricter.

For us, the authenticity dynamic therefore seems to incorporate simultaneous differentiation and integration of the self in relation to what others expect of your appointed role in any particular context. What is, however, important is the person's acceptance of these roles as being all part of the self and thus constituting a whole self. Dissonance between self and a particular role does not result in feeling authentic. When the individual experiences a sense of comfort within a particular role or context, a sense of personality–context fit, the self is affirmed and the authenticity experience enhanced. For us, this again affirms the notion that the authenticity experience is intricately linked to identity work. Whilst most managers seemed more comfortable in their roles at home, one manager for example felt more comfortable at work than at home:

The family always perceive me as someone who's quiet and accommodating … I don't know I just portray that personality at home. In fact they'll often perceive me as someone that will not say anything much. I won't argue a point or whatever, I let it go. But yet in the work environment it's like I feel free to like tell maybe the staff, you know what I think you are wrong and they come to respect that … I think it's really now that I am truly myself and I feel that I am more truly myself in the workplace than I am in more personal circumstances, let's say with my parents-in-law or my family. It's here that there is a certain side of me that they don't see, you know the part that can make decisions, that part of me.

Behaviour regarded as appropriate to the context and the adoption of multiple roles seems to be internally experienced by managers as part of authentic self-expression. Being able to identify appropriate behaviour and adopt multiple roles facilitated authentic behaviour for them. From this, we found that individuals are multidimensional, complex beings who adjust and adapt their behaviour as required. Being true to oneself lies in understanding who you are in relation to external expectations and within the multiplicity of roles that are adopted. Authentic integration affirms the complex yet acceptable multidimensional nature of the self and confirms one's identity as it evolves. Fulfilling multiple roles and adjusting to contextual expectations are not experienced as inauthentic by managers who are able to differentiate and integrate these roles into their identity.

#### Self-confidence facilitates authentic self-expression, which in turn builds self-confidence

When reflecting on a time when they did not feel authentic, various managers alluded to an underlying lack of self-confidence, uncertainty, and heightened self-consciousness at the time. A male manager experienced a lot of uncertainty and distrust whilst he was working under a senior manager whom he did not politically or culturally identify with. “I felt that I was very timid, strained and totally not standing up to this person and in that way I couldn't be myself at all.” Similarly, another experienced uncertainty and could not freely express his true self when starting new in a department with people he did not know or relate to on a social (cultural) level: “if you don't know people or how they feel about you, then you don't quite know how to be with them.” In contrast, self-confidence clearly facilitates the authentic experience and the ability to express oneself without being over-conscious of other people,

I used to be very conscious of what people think of me and everything and now I feel I don't have to go out and prove anything to anybody, so I'm fine actually the way I am,

And “I feel I have this confidence in me, and I don't have to prove to other people what I am or what I can be.” Self-confidence facilitates authentic self-expression, and that again confirms and builds self-confidence, affirming our contention that authenticity evolves to potentially become more stable as one matures and adjusts to find a balance between external expectations and the true self. Self-confidence, however, also seems directly linked to one's position of power in relation to others, and authenticity seems to have come more easily to the managers when they have moved through the ranks and find themselves in their current managerial roles with more authority and power.

I mean there were certain instances where I would and it's curious that when I interact with this person now it's like so different because that person doesn't have that power and is interacting with you on a different level and you are more confident of yourself and can be who you always were.

### Interpretation and discussion

The study aimed to construct an understanding of what it means to be authentic for senior public service managers who are managing diverse teams. The participants were all senior managers who had been leading diverse teams of employees for at least 8 years. Two interrelated themes elucidating the experience of authenticity were found, and we use them to describe authenticity as an intuitive, emotively driven appraisal process of self-congruence in relation to others, and as an experience which incorporates a differentiated yet integrated self.

Our results have led us to conclude that authenticity is inextricably linked to identity work. Research by Heppner et al. (2008) shows significant relationships between self-esteem, need satisfaction, and authenticity, contributing to the authors’ conclusion that with need satisfaction, authenticity may serve to preserve and strengthen one's identity. Authenticity seems to entail an ever-evolving subjective *construction*, *expression*, and *affirmation* of the *self* in that being authentic constitutes an emotively driven and intuitive appraisal process of the extent to which self-expression is experienced as congruent to a perspectival self in context. Humanistic psychological perspectives have long emphasized self-congruent behaviour as key to being authentic (Lenton, Bruder, Slabu, & Sedikides, 2012; Rogers, 1961), and it remains core to more recent authenticity trait theories (see Wood et al., 2008). Whereas Rogers emphasizes congruence between the private self-concept and immediate interpersonal experiences, psychological perspectives on authenticity differ on what constitutes the real self (Mateeva & Dimitrov, 2013). Erickson (1995) refers to upholding self-values, and Maslow (1968) equates the true self to actualizing one's true potential and higher order psychological needs (Goldman & Kernis, 2002; Mateeva & Dimitrov, 2013). Self-determination theorists relate authenticity to behavioural congruence with the intrinsic basic psychological needs of competency, autonomy, and relatedness (Deci & Ryan, 2000; Ménard & Brunet, 2011), and Wood et al. (2008) set actual physiological states, emotions, and deep-level cognitions as self-congruence criteria.

Managers in this study referred to various aspects of the self against which congruence was appraised, including feelings, personality, and values. Authenticity literature mostly refers to operationalizing one's *true self* in your daily interactions (Goldman & Kernis, 2002; Heppner et al., 2008); for us, a sense of a socially constructed *ideal self* emerged that manifests in the perspectival reality of the person and develops as the person matures and adjusts in various work–life contexts. We propose an ideal self because it seemed to us that managers judged their behaviour against a self-benchmark (their perspective of how they should be) which is individually determined by the socially constructed normative perspective of the person. The role of the ideal self in the appraisal dynamic is similarly emphasized by Lenton et al. (2012), who state that people feel authentic because they conform to their own view of an ideal self rather than because they conform to social norms. Erickson (1995) describes authenticity primarily as one's relationship with oneself, without substituting self-attributions with others’ viewpoints. However, managers’ frequent reference to the consistent influence of their social relations on the construction of the self is undeniable, and thus the self both shapes and is shaped by social exchanges. Congruent to a social-constructionist orientation (cf. Crotty, 2005), it is our contention that social norms do impact authenticity as the ideal self is constructed by the individual-in-society. Our position may slightly divert from Heidegger's existentialist notion of *Dasein* (see Duarte, 2005; Escudero, 2013) in that we agree that the authenticity experience is a socially constructed perspectival one but thus pose that the social conformist may also experience being authentic if such self-expression is congruent to the constructed true self. In criticizing the authenticity perspective of evading conformity and affirming individuality, Fletcher (2013) agrees that people can be authentic in retaining their individuality whilst also acting as social agents in a relational context. Several managers presented with concerns regarding the impressions they made on others, linking to the experience of inauthenticity when they felt judged or labelled. In this sense, the self-appraisal process underlying the authenticity experience stands out, and from a psychodynamic perspective ego-defensive processes such as projection may explain managers’ rejection of critique against the self.

Mateeva and Dimitrov (2013) highlight the significant roles of self-knowledge and self-reflective capacity in being authentic. The process of self-appraisal that we propose may be similar to such critical self-reflection through which one is able to be authentic by being conscious of the self, others, relationships, and context (cf. Cranton & Carusetta, 2004; Fletcher, 2013).

Although authenticity is seen here primarily as a subjective phenomenon, subjective experience and construction always happen within and are influenced by context. Research concerning the development of authenticity reveals that factors that influence authenticity begin in early childhood (Harter, 2005). According to Lerner (1993), differing contexts shape individual behaviour, and the work that people engage in creates who they are. Authenticity is thus socially constructed, with one's internal value system and sense of self being developed from being born and brought up in a specific culture. In light of Heidegger's notion of inauthenticity as a result of socialized conformity (see Duarte, 2005), we found that managers are only vaguely aware of the internal tensions created by their need for belonging and thus wanting to conform versus claiming a unique identity. These tensions give rise to an affective state, through which managers then appraise the self as either being authentic or not.

We found it very interesting that the managers seemed unaware of this internal appraisal dynamic, and therefore we infer that the process is an intuitive one. The self-appraisal process seems to manifest in a recurring fashion in that you continuously and instinctively appraise your behaviour, and when positive emotions result, authenticity is experienced and the self is affirmed. The authenticity experience surfaces more readily to a conscious level when one becomes aware of negative emotions in relation to the self in a particular context giving rise to critical self-reflection. Self-congruence seems to be cognitively and emotively judged as appropriate or not, yet it is the affective consequence that brings the authenticity experience to conscious awareness. Our findings resonate with Lenton et al.'s (2012) distinction between state authenticity and trait authenticity. State authenticity is defined as a phenomenological experience of the self in which being authentic is felt rather than cognitively evaluated. Vannini (2006) also emphasized that individuals feel strong positive emotions when they experience congruence with their values, goals, emotions, and self-meaning.

Overall, self-appraisal seems to consistently and intuitively occur, and as the process continues throughout the work–life span, one's identity is affirmed, and we propose that the experience of being authentic evolves and may become more stable through time. Mateeva and Dimitrov (2013, p. 205) confirm the temporal and intuitive nature of the authentic experience: “Rather, authenticity may be conceptualised as an on-going psychodynamic process whereby one's potentials, characteristics, emotions, values, motivation, and so forth are discovered and explored, accepted, imbued with meaning or purpose and expressed and actualised.”

As part of this on-going, evolving self-authentication process, we propose that the authenticity experience incorporates a dynamic process of self differentiation and integration.

The managers in this study reported that their work role differed from other roles. The work context required them to adopt different behaviours that were seen by them to be contextually appropriate. The workplace may thus be an important contributor to shaping individual identity and consequently one's experience of authenticity. All of the managers regarded behavioural adjustment and self-differentiation across roles and contexts as an appropriate expression of their being authentic. Managers demonstrated an awareness and willingness to integrate multiple roles and to display and express self differently across contexts without feeling inauthentic. One could deduce that such integration is driven by their need for social acceptance and belonging. Although managers clearly feel more comfortable in certain contexts or roles, the ability to differentiate and integrate is experienced as being authentic as it provides them the freedom to choose how they are where and with whom. Kernis (2003) views the authentic individual as responsive to situational and role requirements and authenticity as an on-going process through which both differentiation and integration of the self-manifest. Research suggests that we do not have one true self and that multiple selves are not experienced as inauthentic, but rather as necessary for interaction and for the preservation of relationships (Kernis, 2003; Lerner, 1993). Erickson (1995) qualifies authenticity and inauthenticity with the adjective *relative*, implying that authenticity is not an either/or condition; that is, people are never entirely authentic or inauthentic, but they can be described as achieving levels of authenticity (Avolio & Gardner, 2005; Harvey, Martinko, & Gardner, 2006). As such, it can be reasoned that leaders can become increasingly authentic over time (see also Harvey et al., 2006). From the managers’ viewpoint, achieving authentic differentiation and integration of the self is facilitated by developing self-confidence. Similar to the reciprocal effect between authenticity and self-esteem described in the study by Heppner et al. (2008), our data demonstrate that self-confidence facilitates authentic self-expression, and in turn authentic self-expression builds self-confidence. Built into the ideal of authentic living is the idea that a unique self is distinguished by the individual's thoughts, feelings, needs, desires, capacities, aptitudes, beliefs, and preferences (Guignon, 2004) and that self-knowledge, awareness, and self-understanding precede the ability to live and act according to the unique self (Guignon, 2004; Harter, 2005; Kernis, 2003). In this study's context, the political work context and the power position of the individual in particular affected managers’ authenticity experiences as their need for belonging is particularly challenged by opposing political agendas. In this context, managers are frequently confronted by their ontological anxiety (cf. Heidegger, 1962), and coping is exemplified in those who are able to differentiate and integrate the self within the work context. Self-perception and acceptance rise according to the extent to which individuals justify their place in power relations and political in-groups, and a stronger power position seems to result in a heightened sense of being authentic. In essence, this would imply inauthenticity from Heidegger's point of view, yet research by Kifer et al. (2013) provides consistent evidence that having power enhances the authenticity experience and results in an increased subjective well-being. Linking to the effects of intergroup processes and intragroup dynamics on individuals’ authenticity dynamics (argued by Mateeva & Dimitrov. 2013), in this study's context, we also found managers who related authentic experiences more readily as their hierarchical position in a politicized organizational structure improved and they consequently felt more assured of their role in the in-group. We further found that feeling authentic eluded those individuals who could not differentiate a unique self whilst also conforming to role and contextual expectations. In particular, this was evidenced in some role expectations that could not be integrated with personal affirmation. Again, whether such conforming constitutes true authentic living is not for us to say, as we believe the authentic experience lies in the perspectival reality of the individual. Ultimately, our interpretation of the data led us to believe that authenticity is part of the dynamic process of identity formation, as the self constantly endeavours to affirm itself in relation to others and by integrating adjusted self-roles appropriate to contextual expectations without losing a sense of unique self in the process.

## Methodological considerations

The aim of this study was to understand and describe the meaning of authenticity for senior managers in public service, in order to gain understanding of the authenticity dynamics prevalent in their everyday work life. We have aligned our driving epistemological assumptions and methodological notions with interpretative phenomenology. A rich description emanated from decontextualizing and recontextualizing the managers’ lived experience of being authentic. We strived to maintain an open attitude throughout the study by making our preconceptions explicit when formulating the rationale and aim of the study and critically thinking about who we are as primary research instruments directing this study (reflexive bracketing). Phenomenological reduction was further strived for by attempting an initial naïve immersion in the text followed by a natural account of the meaning derived from managers’ experience of authenticity. Integrating theory with meaning clusters from the data in a transparent manner was conducted as part of the final critical interpretation phase. Due to the inherent subjective nature of qualitative research and our belief that it is not fully possible to set aside one's preconceptions (cf. Norlyk & Harder, 2010), we only claim to make a contribution to the existing conceptual literature on authenticity. In particular, the findings here only shed light on being authentic as experienced by senior managers in a diverse, politically driven, public service work context.

## Conclusion

Apart from limited research on the construct of authenticity, studying the experience of authenticity enhanced our understanding of managers’ ability to cope with challenges of identity work in the workplace. We believe that doing affirmative identity work with managers will enhance their authenticity experience. We further believe that feeling authentic enhances the manager's sense of self in relation to others and thus enables subjective well-being, assertive behaviour, building of constructive relationships, and confident decision making in a politically laden work environment.
